# *Pectus* iatrogênico após esternotomia em pacientes com síndrome de Down

**DOI:** 10.1055/s-0045-1809524

**Published:** 2025-07-10

**Authors:** Davi de Podestá Haje, Jorge Henrique Carlos Aires, Talita Virginia Pinto de Sousa, Fernando Aurélio de Sá Aquino

**Affiliations:** 1 Centro Clínico Orthopectus, Brasília, DF, Brasil; 2 Departamento de Cirurgia Ortopédica, Hospital de Base do Distrito Federal, Brasília, DF, Brasil

**Keywords:** deformidades, doença iatrogênica, esternotomia, pectus carinatum, pectus excavatum, síndrome de Down, deformity, Down syndrome, iatrogenic disease, pectus carinatum, pectus excavatum, sternotomy

## Abstract

**Objetivo:**

Avaliar a incidência de deformidade *pectus* após esternotomia em pacientes com síndrome de Down e suas características clínicas e radiográficas.

**Métodos:**

Foram estudados 20 pacientes com histórico de esternotomia durante a infância e um grupo controle ( *n* = 20). O tórax foi avaliado clinicamente quanto à presença e tipo de deformidade *pectus* , gravidade e comprimento clínico do corpo esternal. Exames radiográficos foram utilizados para avaliar quaisquer anormalidades.

**Resultados:**

Do total, 85% ( *n* = 17) apresentaram deformidades *pectus* (41% *carinatum* lateral, 65% gravidade branda e 29% pouca flexibilidade). No grupo controle, a deformidade ocorreu em 5% ( *n* = 1). No grupo esternotomia, 40% ( *n* = 8) apresentaram esterno clinicamente encurtado, o que não ocorreu no grupo controle ( *p* = 0,01). O exame radiográfico do grupo esternotomia com *pectus* mostrou angulações posteriores no manúbrio (10%), encurtamento esternal (38%) e irregularidades no corpo esternal (70%); além disso, 36% das crianças tinham todas as placas de crescimento esternal fechadas e 10% apresentaram fusão esterno-manubrial precoce, o que não ocorreu no grupo controle.

**Conclusão:**

Os pacientes apresentaram uma alta incidência de deformidade *pectus* após esternotomia (principalmente leve e do tipo *carinatum* lateral), com alterações radiográficas sugestivas de crescimento esternal anormal.

## Abstract (en)

**Objective:**

To evaluate the incidence of pectus deformity after sternotomy in patients with Down syndrome, in addition to the clinical and radiographic characteristics.

**Methods:**

There were 20 patients with sternotomy history during childhood and a control group ( *n* = 20) were studied. The chest was clinically evaluated for the presence and type of pectus deformity, severity, and the clinical sternal body length. Radiographic examinations were used to evaluate any abnormalities.

**Results:**

From the total, 85% ( *n* = 17) presented with pectus deformities (41% lateral pectus carinatum, 65% mild severity, and 29% little flexibility). In the control group, deformity occurred in 5% ( *n* = 1). In the sternotomy group, 40% ( *n* = 8) had a clinically shortened sternum, which did not occur in the control group ( *p* = 0.01). Radiographic examination of the sternotomy with pectus group showed posterior angulations in the manubrium (10%), sternal shortening (38%), and irregularities in the sternal body (70%); furthermore, 36% of the children had all sternal growth plates closed, and 10% had early sterno-manubrial fusion, which did not occur in the control group.

**Conclusion:**

Patients presented a high incidence of pectus deformity after sternotomy (mostly mild and of the lateral carinatum type), with radiographic changes suggestive of abnormal sternal growth.

## Introdução

As deformidades *pectus* são idiopáticas na maioria dos casos, mas também podem ser iatrogênicas, 1 2 congênitas ou patológicas (por exemplo, síndrome de Marfan). 3 Essas deformidades são observadas principalmente por volta dos 10 anos de idade e tendem a piorar durante a puberdade. 4

O crescimento do esterno ocorre por ossificação endocondral 5 6 das placas de crescimento entre seus segmentos ósseos e as junções costocondrais, como originalmente descrito por Haje e Bowen. 4 O início de distúrbios do crescimento esternal durante os períodos pré-natal e de desenvolvimento pode gerar uma desproporção entre o esterno e as costelas.

Haje et al. 7 descreveram que lesões nas placas de crescimento esternal de ratos podem gerar deformidades *pectus* . Lesões primárias ou distúrbios anatômicos das placas de crescimento esternal por esternotomia à cirurgia cardíaca parecem causar deformidades durante o período de crescimento das crianças. 2 8 9 Outra possível causa seria a persistência de uma lacuna macroscópica entre as placas de crescimento ou as duas metades do corpo esternal após o fechamento da esternotomia. 9

As radiografias em perfil revelaram fechamento precoce das placas de crescimento esternais em pacientes com *pectus* , especialmente do tipo *carinatum* superior ou Currarino, gerando corpos esternais encurtados e curvos. 10

O primeiro relato de caso de deformidade *pectus* iatrogênica é de 1995 e se refere a *pectus carinatum* pós-esternotomia para tratamento de malformação cardíaca. O paciente foi tratado satisfatoriamente com a órtese Dynamic Chest Compressor 1 (DCC 1), 8 descrita pela primeira vez por Haje e Raymundo em 1979. 11 Outro caso de *pectus carinatum* iatrogênico foi descrito. 12 No entanto, o número de casos pode ser subnotificado, especialmente aqueles com deformidade branda que podem não ser priorizados pelos médicos ou familiares.

Haje et al. 9 também classificaram as deformidades *pectus* idiopáticas em termos de tipo clínico, gravidade e flexibilidade; 1 4 9 no entanto, esses fatores não foram analisados no contexto de deformidades *pectus* iatrogênicas.

O objetivo deste estudo foi avaliar a prevalência de deformidades *pectus* iatrogênicas em pacientes esqueleticamente imaturos com síndrome de Down submetidos à esternotomia durante o tratamento cirúrgico de cardiopatias congênitas. Os objetivos secundários foram avaliar o tipo de deformidade, sua flexibilidade, gravidade e as alterações radiográficas nestes pacientes.

## Materiais e Métodos

Os dados clínicos para a montagem dos grupos submetidos ou não à esternotomia foram coletados dos prontuários dos pacientes com síndrome de Down. Todos os responsáveis legais foram informados sobre o estudo e assinaram o termo de consentimento livre e esclarecido. O protocolo de avaliação foi aprovado pelo Comitê de Ética Institucional (37229014.0.1001.5553).

**Fig. 1:**
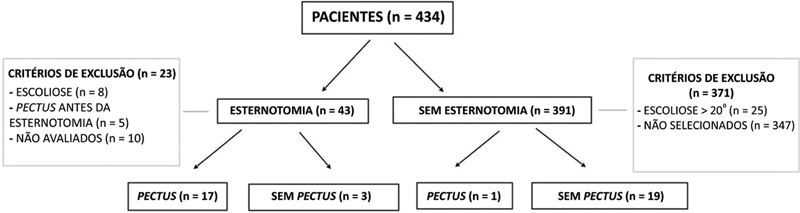
Fluxograma de seleção de pacientes para os grupos de estudo (grupos esternotomia e controle).

O estudo incluiu 434 prontuários de pacientes com síndrome de Down, dos quais 43 tinham histórico de esternotomia na infância para reparo cardíaco. A Fig. 1 mostra o fluxograma de seleção de pacientes para o estudo. Foram 20 pacientes compondo o grupo esternotomia (8 do sexo masculino e 12 do feminino; idade média: 12,51, intervalo: 1–24 anos, desvio-padrão [DP]: 7,16) dos quais 11 eram crianças ou adolescentes em crescimento e 9 eram adultos. Dos 391 pacientes restantes, 20 sem histórico de esternotomia foram selecionados aleatoriamente como grupo controle (8 do sexo masculino e 12 do feminino; idade média: 14,67 anos, intervalo: 1–34 anos, DP: 9,04), com 11 crianças ou adolescentes em crescimento e 9 adultos.

A idade média dos pacientes à esternotomia foi de 24,35 meses. O paciente mais velho no momento da esternotomia tinha 96 meses.

As avaliações clínicas no ambulatório de ortopedia começaram após a alocação dos pacientes em grupos.

Os familiares dos pacientes foram questionados sobre a data da esternotomia anterior, a presença de deformidade *pectus* antes da operação, o tempo de início do *pectus* após, o histórico familiar de deformidades torácicas e o tratamento do *pectus* anterior. As avaliações clínicas foram realizadas por um único ortopedista pediátrico especializado no tratamento não invasivo de deformidades torácicas. As análises incluíram o tipo de deformidade torácica, a gravidade (branda, moderada ou grave), a flexibilidade, o comprimento do esterno (normal ou encurtado, este último quando o esterno ou processo xifoide terminava proximalmente à linha do mamilo) e a presença de cifose torácica exacerbada ou escoliose.

As deformidades foram classificadas como *pectus carinatum* superior (PCS), *pectus carinatum* inferior (PCI), *pectus carinatum* lateral (PCL), *pectus excavatum* amplo (PEA) e *pectus excavatum* localizado (PEL), conforme descrito em estudos anteriores. 1 10 11 13

**Fig. 2:**
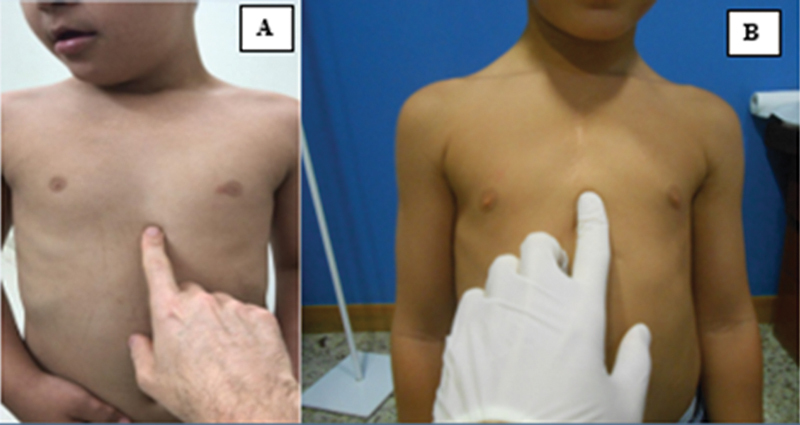
Avaliação do comprimento do esterno em um paciente com esterno de comprimento normal ( **A** ) e em um paciente com esterno encurtado ( **B** ).

A avaliação do comprimento do esterno é mostrada na Fig. 2 . foi avaliada pela compressão manual do ápice da deformidade em direção anteroposterior. A flexibilidade do *pectus excavatum* foi avaliada pela compressão manual das protrusões do rebordo costal inferior em direção anteroposterior, com o paciente simultaneamente realizando uma manobra de Valsalva com adução do braço contra resistência, e observação dos efeitos na área de depressão. 4 Os pacientes que não conseguiam realizar a manobra de Valsalva foram orientados a soprar um balão ou foram isentos deste componente de avaliação.

Tanto *pectus carinatum* quanto *excavatum* foram classificados como muito ou moderadamente flexíveis em caso de reversão significativa ou completa da deformidade, respectivamente. Alternativamente, a deformidade foi classificada como menos flexível quando apresentava pouca alteração, ou rígida em caso de nenhuma com as manobras realizadas.

Os achados de imagem do tórax de todos os pacientes dos grupos de esternotomia e controle foram documentados. As radiografias foram obtidas em projeção em perfil e oblíqua do esterno.

**Fig. 3:**
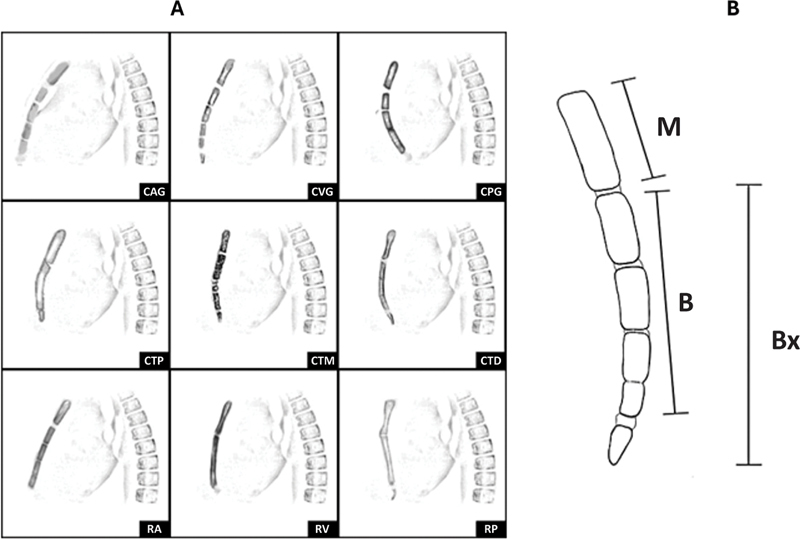
( **A** ) Ilustração dos padrões esternais: curvatura anterior gradual (CAG), curvatura vertical gradual (CVG), curvatura posterior gradual (CPG), curvatura do terço proximal (CTP), curvatura do terço médio (CTM), curvatura do terço distal (CTD), curvatura retilínea anterior (RA), curvatura retilínea vertical (RV) e curvatura retilínea posterior (RP) 14 . ( **B** ) Diagrama de medidas obtidas da projeção em perfil do esterno de uma criança com tórax normal e idade aproximada de 10 anos, com duas placas de crescimento abertas. O comprimento do manúbrio (M) e do corpo do esterno (B) e a linha reta traçada da extremidade proximal do corpo esternal até a extremidade distal do xifoide (Bx) foram medidos em centímetros. O resultado obtido pela divisão do comprimento do corpo esternal (B) pelo comprimento do manúbrio (M) foi chamado de índice BM. Quando o processo xifoide estava ossificado, o índice BxM foi medido, representado pela divisão de Bx por M 5 .

A presença de irregularidade laterolateral do corpo esternal foi avaliada em projeção oblíqua. 9 Os parâmetros radiográficos observados na projeção em perfil foram o número de placas de crescimento esternal abertas (0 a 3), número total de suturas na placa de crescimento, padrão de angulação sagital do esterno, 14 assim como índices corpo esternal-manúbrio (BM) ou xifoide-manúbrio (BxM) ( Fig. 3 ). 5 Um estudo anterior demonstrou que os dois índices foram constantes em pacientes normais, independentemente da idade, com valores variando de 2,16 ± 0,24 (BM) a 2,73 ± 0,31 (BxM). Esses intervalos de normalidade foram aplicados no presente estudo. 5

Outros achados radiográficos anormais foram observados. Embora tomografias computadorizadas (TC) de tórax não tenham sido solicitadas, pacientes que passaram por esse exame por outros motivos tiveram suas imagens analisadas.

Radiografias totais da coluna em posição ortostática foram solicitadas em pacientes com achados clínicos sugestivos de escoliose ou cifose exacerbada para determinação da gravidade da curva pelo ângulo de Cobb.

O teste de Shapiro-Wilk foi utilizado para análise estatística comparativa entre variáveis numéricas de cada grupo que seguiram distribuição normal. Quando a hipótese de normalidade não foi rejeitada, o teste *t* para amostras independentes foi utilizado para comparação entre os grupos. Em caso de rejeição da hipótese de normalidade, o teste não paramétrico de Mann-Whitney foi usado. O teste qui-quadrado (x ^2^ ) foi empregado para comparação de variáveis categóricas entre os grupos.

As variáveis número de placas de crescimento esternal abertas (0–3) e presença de suturas não foram comparadas em nenhum dos grupos, por estarem relacionadas à idade e porque o número de crianças e adolescentes em ambos os grupos era insuficiente para uma análise estatística.

## Resultados

No grupo de 20 pacientes submetidos à esternotomia, 85% ( *n* = 17) apresentaram deformidades *pectus* após o procedimento. O tempo médio de início foi de 5 meses (mediana = 3 meses), com 25% dos casos ocorrendo logo após a cirurgia ( *n* = 5), 15% ( *n* = 3) em 3 meses, 5% ( *n* = 1) em 4 meses, 5% ( *n* = 1) em 6 meses e 5% ( *n* = 1) em 36 meses. Em 30% ( *n* = 6) dos casos, os familiares não conseguiam se lembrar.

A idade média de início do *pectus* neste grupo foi de 27,47 meses (2,29 anos).

No grupo controle, o único paciente com deformidade apresentou *pectus excavatum* localizado aos 6 meses de idade, com gravidade leve e flexibilidade moderada. Nenhum paciente de ambos os grupos tinha histórico familiar de deformidades *pectus* .

A Tabela 1 mostra os tipos de *pectus* , sua gravidade e flexibilidade no grupo esternotomia.

**Tabela 1:** Tipos de *pectus* , gravidade e flexibilidade no grupo esternotomia

Tipo de *pectus*	Gravidade			Flexibilidade		
	Branda	Moderada	Grave	Moderada ou alta	Rígida ou baixa	Total
PCI	11,8% ( *n* = 2)	5,9% ( *n* = 1)	11,8% ( *n* = 2)	23,6% ( *n* = 4)	5,9% ( *n* = 1)	29,5% ( *n* = 5)
PCL	29,5% ( *n* = 5)	11,8% ( *n* = 2)	0,0% ( *n* = 0)	35,3% ( *n* = 6)	5,9% ( *n* = 1)	41,1% ( *n* = 7)
PCS	5,9% ( *n* = 1)	0,0% ( *n* = 0)	0,0% ( *n* = 0)	0,0% ( *n* = 0)	5,9% ( *n* = 1)	5,9% ( *n* = 1)
PEL + PCL*	0,0% ( *n* = 0)	5,9% ( *n* = 1)	0,0% ( *n* = 0)	0,0% ( *n* = 0)	5,9% ( *n* = 1)	5,9% ( *n* = 1)
PEL	17,6% ( *n* = 3)	0,0% ( *n* = 0)	0,0% ( *n* = 0)	11,8% ( *n* = 2)	5,9% ( *n* = 1)	17,6% ( *n* = 3)
Total	64,8% ( *n* = 11)	23,6% ( *n* = 4)	11,8% ( *n* = 2)	70,7% ( *n* = 12)	29,5% ( *n* = 5)	100,0% ( *n* = 17)

**Abreviaturas:** PCI, *pectus carinatum* inferior; PCL, *pectus carinatum* lateral; PCS, *pectus carinatum* superior; PEL, *pectus excavatum* localizado.

**Nota:** * *Pectus* de tipo misto.

A escoliose branda foi encontrada em 40 ( *n* = 8) e 20% ( *n* = 4) dos pacientes dos grupos esternotomia e controle, respectivamente, com diferença estatística ( *p* < 0,001). Todos os pacientes dos dois grupos apresentavam cifose postural.

**Fig. 4:**
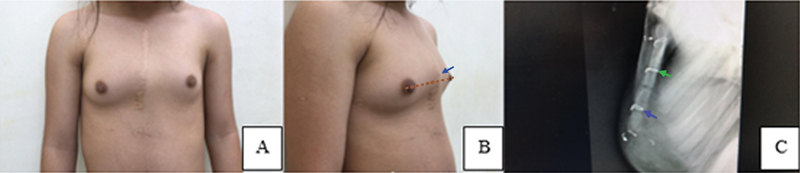
( **A** ) Projeção anterior de paciente de 13 anos submetido à esternotomia aos 18 meses de idade. A projeção oblíqua direita revela *pectus carinatum* inferior (PCI). ( **B** ) A extremidade distal do esterno ou xifoide (ápice da deformidade – seta) era proximal à linha do mamilo (linha tracejada), demonstrando o encurtamento clínico deste osso. ( **C** ) A projeção em perfil do esterno revela a angulação posterior do osso manúbrio (seta 1), a sutura metálica na junção esterno-manubrial com fusão incipiente (seta 2), duas fises de crescimento abertas, uma sutura metálica na fise de crescimento distal (seta 3) com padrão esternal retilíneo e índice BM < 2.

Em todos os pacientes do grupo controle, o comprimento esternal era clinicamente normal, enquanto 40% ( *n* = 8) do grupo submetido à esternotomia apresentavam esterno clinicamente encurtado; diferenças significativas foram observadas entre os grupos ( *p* = 0,01). A Fig. 4 mostra um paciente do grupo esternotomia com PCI e terminação do esterno à altura mamilo, representando encurtamento esternal ( Fig. 4A,B ); a projeção radiográfica em perfil do esterno revela diversas alterações ( Fig. 4C ).

**Fig. 5:**
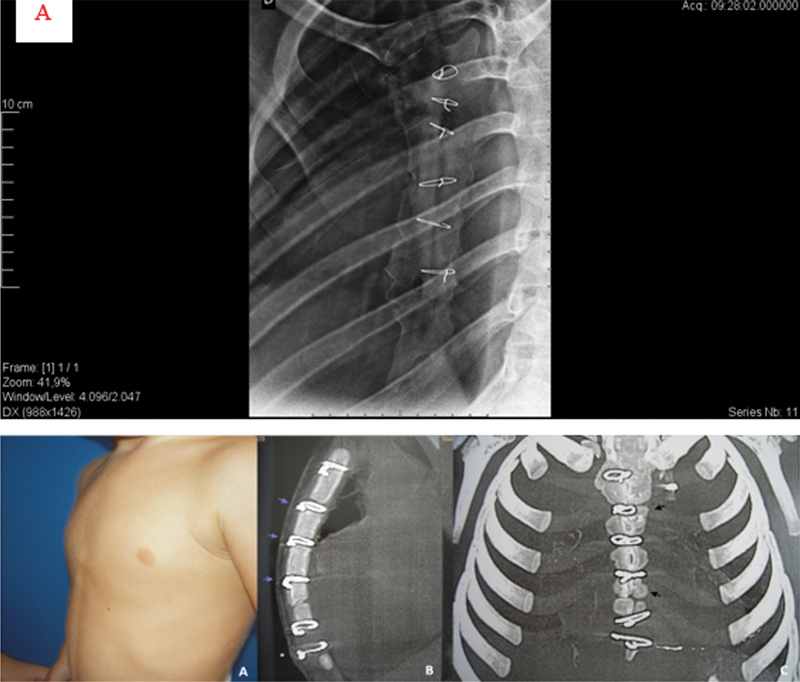
( **A** ) Projeção oblíqua de um paciente de 24 anos com *pectus carinatum* lateral brando submetido à esternotomia aos 96 meses de idade, com fusão esterno-manubrial e irregularidades laterolaterais (assimetrias nos contornos laterais nos dois lados do esterno). ( **B** ) Imagem de tomografia computadorizada de um paciente de dois anos com *pectus carinatum* inferior (PCI) submetido à esternotomia ( **a** ). Imagem sagital mostrando estruturas metálicas nas duas placas de crescimento proximais e na junção esternomanubrial, com padrão esternal de CPG (setas) ( **b** ). Imagem coronal reformatada mostrando placas de crescimento proximais irregulares e irregularidade ou assimetria laterolateral na comparação dos dois lados externos (setas) ( **c** ).

A medida do índice BM mostrou comprimento esternal normal em todos os pacientes sem histórico de esternotomia. Em 38% ( *n* = 6) do grupo esternotomia, os índices BM e BxM revelaram esternos radiograficamente encurtados, em proporção significativamente em comparação ao grupo controle (38 vs. 0%, *p* = 0,05). A avaliação radiográfica oblíqua do esterno mostrou que as irregularidades laterolaterais foram significativamente mais comuns no grupo esternotomia (70 vs. 20%, *p* = 0,01), como notado na Fig. 5 .

Suturas metálicas em placas de crescimento foram encontradas em pacientes submetidos à esternotomia, ainda em crescimento e com placas de crescimento esternais ainda abertas ( *n* = 11). Destes, 36% ( *n* = 4) apresentavam suturas nas placas de crescimento: dois pacientes tinham suturas em duas placas de crescimento e dois pacientes, em uma placa de crescimento. Um desses pacientes foi submetido à TC em outro hospital ( Fig. 5 ). Todos os pacientes sem deformidades *pectus* no grupo esternotomia ( *n* = 3) já eram adultos durante a avaliação radiográfica, impossibilitando a determinação da colocação prévia de suturas metálicas nas placas de crescimento. Os índices BM e BxM, a presença de irregularidades laterolaterais e o número de placas de crescimento esternais abertas nos grupos de esternotomia e controle são detalhados na Tabela 2 .

**Tabela 2:** Resultados do índice corpo esternal-manúbrio (BM)

Achados radiográficos		Grupo		
		Controle	Esternotomia	Total
**Índice BM / índice BxM**	<2,16 ou < 2,73*	0% ( *n* = 0/15)	38% ( *n* = 6/16)** ^s^	20% ( *n* = 6/31)
	<2,16 ou < 2,73*	100% ( *n* = 15/15)**	63% ( *n* = 10/16)** ^s^	80% ( *n* = 25/31)
**Irregularidade laterolateral**	Sim	20% ( *n* = 4/20)	70% ( *n* = 12/17) ***	43% ( *n* = 16/37)
	Não	80% ( *n* = 16/20)	30% ( *n* = 5/17) ***	57% ( *n* = 21/37)
**Abertura das placas de crescimento** ****	1	15% ( *n* = 3/20)	0% ( *n* = 0/20)	7,5% ( *n* = 3)
	2	10% ( *n* = 2/20)	10% ( *n* = 2/20)	10% ( *n* = 4)
	3	30% ( *n* = 6/20)	20% ( *n* = 4/20)	25% ( *n* = 10)
**Placas de crescimento fechadas**		45% ( *n* = 9/20) adultos 0% ( *n* = 0/20) crianças ^&^	45% ( *n* = 9/20) adultos 25% ( *n* = 5/20) crianças ^&^	57,5% ( *n* = 23)

**Abreviaturas:** BM, índice corpo esternal-manúbrio; BxM, índice xifoide-manúbrio. **Notas:** * < ou > 2,16 valores relativos ao índice BM e < ou > 2,73 valores relativos ao índice BxM 5 . ** Cinco pacientes foram excluídos por apresentarem fusão esterno-manubrial que impedia a mensuração dos índices BM ou BxM. ** ^s^ Quatro pacientes foram excluídos por apresentarem fusão esterno-manubrial que impedia a mensuração dos índices BM ou BxM. *** Falha de análise em três pacientes submetidos à esternotomia devido a radiografias de má qualidade. **** Todos os pacientes com placas de crescimento abertas eram crianças. ^&^ Os pacientes com todas as placas de crescimento fechadas, 0% (n = 0/9) e 36% (n = 5/14), eram crianças do grupo controle e esternotomia, respectivamente.

No grupo esternotomia, dois pacientes (10%) apresentaram angulação posterior no terço médio do manúbrio (o que não ocorreu no grupo controle), um deles associado à ossificação na região anterior, 20,0% ( *n* = 4) apresentaram fusão esterno-manubrial (dois pacientes eram crianças com deformidades *pectus* e os demais eram adultos, um sem deformidade) e 10,0% ( *n* = 2) apresentaram outra angulação aguda no terço distal do esterno, sendo um com angulação anterior e posterior. No grupo controle, 25% ( *n* = 5) pacientes, todos adultos, apresentaram fusão esterno-manubrial.

Acerca do tipo de curvatura esternal, no grupo esternotomia, 25% ( *n* = 5) dos pacientes tinham GAC, 5% ( *n* = 1), curvatura gradual posterior, 30% ( *n* = 6), CVG, 25% ( *n* = 5), RA, 5% ( *n* = 1) RP e 10% ( *n* = 2), curvatura retilínea vertical. No grupo controle, 50% ( *n* = 10) apresentaram CVG, 40% ( *n* = 8), RA e 10% ( *n* = 2), RP. O teste X ^2^ não revelou diferenças significativas quanto ao tipo de angulações sagitais entre os grupos ( *p* = 0,20).

**Fig. 6:**
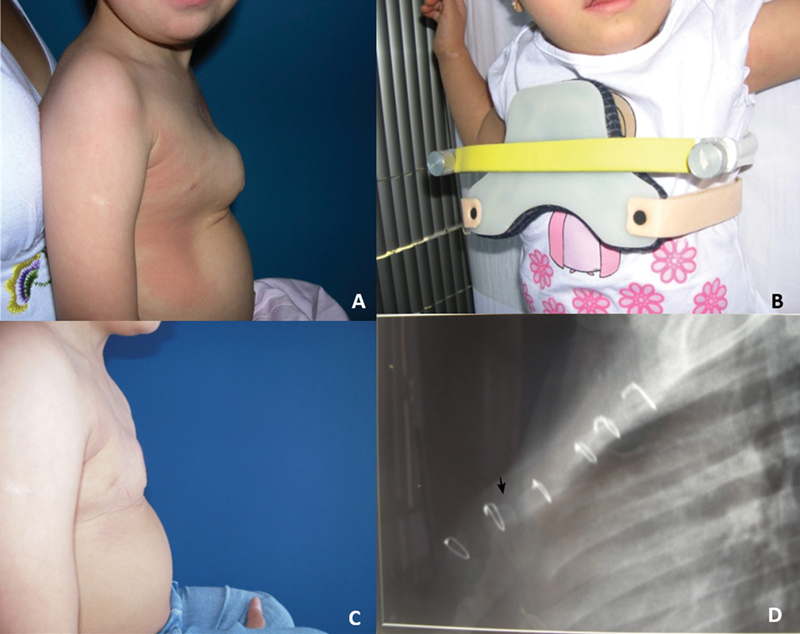
Paciente portador de síndrome de Down submetido à esternotomia para correção cardíaca aos 6 meses de idade e novamente aos 13 meses de idade apresentou ( **A** ) *pectus carinatum* inferior grave aos 2 anos e 1 mês de idade, ( **B** ) quando iniciou o uso de órtese DCC 1, ( **C** ) com boa correção parcial aos 24 meses de acompanhamento. ( **D** ) Radiografia em perfil do esterno mostrando padrão retilíneo anterior (RA) com maior angulação anterior aguda em sua porção do terço distal (seta) e fusão esterno-manubrial precoce.

Dos pacientes do grupo esternotomia que desenvolveram deformidades *pectus* , três foram tratados com órtese ( Fig. 6 ).

## Discussão

Este é o primeiro estudo a analisar a prevalência de deformidade *pectus* em pacientes submetidos à esternotomia. Nosso achado de alta prevalência dessas deformidades em pacientes submetidos à esternotomia (85%) propõe que as famílias precisam ser informadas sobre esse risco antes do encaminhamento para cirurgia cardíaca que necessite de esternotomia.

Neste estudo, 25,0% ( *n* = 5) dos pacientes submetidos à esternotomia foram diagnosticados com deformidade *pectus* logo após o procedimento, inferindo uma alteração pós-operatória imediata no osso esternal. Além disso, a idade média de início do quadro foi de 2,5 anos após a esternotomia, muito antes do que os casos idiopáticos, que surgiram principalmente no período pré-adolescente. 4 9

Segundo Ellis, 40% dos pacientes tinham histórico familiar de deformidade *pectus* . 15 Entretanto, isso não ocorreu em nossa amostra, em que nenhum dos pacientes com deformidade *pectus* apresentava histórico familiar, reforçando a etiologia iatrogênica.

O *pectus excavatum* é o tipo mais frequente em estudos de prevalência, 16 embora um estudo de Haje et al., 17 com 4.012 pacientes, tenha relatado que 79% dos pacientes tinham *pectus carinatum* (PCI: 45%, PCL: 28%, PCS: 5%, PEL: 13%, PEA: 9%). 17 A comparação entre esta série de estudos de casos iatrogênicos com os relatos de Haje e Haje 17 mostrou que o PCL foi o tipo predominante de *pectus* , o que pode ser justificado por uma possível aposição irregular entre os dois lados do esterno durante o fechamento cirúrgico do osso.

Haje et al. 5 não mostraram suturas entre os segmentos ósseos do esterno, descritas anteriormente por Currarino e Silverman, 5 18 mas a presença de placas de crescimento. O comprimento do esterno aumenta devido a sua ação. Essas placas também são responsáveis pelo crescimento das cartilagens costais e costelas na parede torácica anterior. Qualquer agressão cirúrgica a essas estruturas pode fazer com que o crescimento seja desproporcional e, assim, causar deformidades. 1 5 7 10 Neste estudo, 70,0% dos pacientes com *pectus* submetidos à esternotomia ( *n* = 12) apresentaram irregularidades laterolaterais, fechamento precoce da placa de crescimento, fusão esterno-manubrial e outras alterações que não foram observadas no grupo controle, como angulação posterior do manúbrio, angulação aguda do terço distal do esterno e ossificação irregular anterior ao corpo esternal.

Em um estudo anterior, TCs com reconstrução coronal mostraram que irregularidades laterais do corpo esternal foram mais frequentes em pacientes com *pectus* do que no grupo controle ( *n* = 10). Além disso, essas irregularidades foram mais difíceis de observar e interpretar em radiografias oblíquas do esterno. No entanto, devido a preocupações com radiação, a TC de tórax para diagnóstico não foi rotineiramente recomendada. 10

A fusão esternomanubrial foi mais frequente em pacientes adultos, mas também ocorreu em dois pacientes pediátricos do grupo esternotomia. De modo geral, essa fusão não ocorre em crianças e é observada apenas em 10 a 30% dos casos na idade adulta, 12 embora tenhamos observado uma incidência de 55% em nossos pacientes adultos do grupo controle.

Este estudo incluiu a avaliação clínica dos comprimentos esternais e 40,0% ( *n* = 8) dos pacientes submetidos à esternotomia apresentaram esterno encurtado, diferentemente do grupo controle, em que não houve tal achado. Não há relatos anteriores do comprimento clínico do esterno na literatura e os autores acreditam que esta medida deva ser incluída no exame físico desses pacientes.

O grupo esternotomia apresentou esternos mais encurtados em radiografias (índice BM anormal) do que o grupo controle, reforçando o conceito de que a agressão cirúrgica às placas de crescimento esternal pode encurtar o osso e gerar deformidades, em sua maioria brandas. O índice BM já foi usado para avaliar o encurtamento radiográfico do esterno, sugerindo que uma desproporção entre os crescimentos esternal e costal pode provocar uma deformidade *pectus* . 5

Este estudo também avaliou o número de placas esternais de crescimento abertas ou fechadas, que estão programadas para fechar de acordo com a faixa etária, com variação individual. No grupo esternotomia, cinco crianças com deformidade *pectus* tinham todas as placas fechadas, o que não ocorreu no grupo controle.

O tratamento das deformidades *pectus* descrito inclui DCCs e correção cirúrgica. 1 4 7 8 9 11 Segundo Haje et al., 7 9 a flexibilidade é o fator prognóstico mais importante no tratamento conservador. Com PCI e PCL idiopáticos sendo os tipos mais flexíveis de *pectus carinatum* , enquanto PCS é mais rígido e resistente ao tratamento ortótico. 9 17 No entanto, neste estudo, dois casos de PCL e PCI apresentaram rigidez durante a infância, uma apresentação incomum. Portanto, as deformidades *pectus* iatrogênicas tendem a ser mais rígidas do que seus correspondentes idiopáticos.

Cientes de que lesões nas placas de cartilagem de crescimento podem causar deformidade em curto e longo prazo, os cirurgiões ortopédicos geralmente tentam preservá-las ao operar ossos longos em crianças e adolescentes. Esse cuidado deve ser estendido às cirurgias realizadas em todos os ossos da região torácica. Logo, técnicas aprimoradas de sutura esternal podem diminuir o aparecimento de deformidade *pectus* , poupando as placas de crescimento esternal ou fazendo sua aposição com a maior precisão possível durante a sutura esternal.

## Conclusão

Concluindo, a esternotomia em pacientes com síndrome de Down foi associada a uma alta prevalência de deformidade *pectus* , em sua maioria brandas, do tipo PCL, com alterações radiográficas.
